# Influence of Different Preactivation Patterns and Aligner Materials on the Capability of Aligners to Induce Palatal Root Torque of Upper Incisors: An In Vitro Biomechanical Study

**DOI:** 10.1111/ocr.12940

**Published:** 2025-05-03

**Authors:** Sophia Weber, Stefan Repky, Rudolf Jäger, Falko Schmidt, Bernd G. Lapatki, Fayez Elkholy

**Affiliations:** ^1^ Department of Orthodontics University of Ulm Ulm Germany; ^2^ Institute of Statistics Ulm University Ulm Germany

**Keywords:** aligner, bodily movement, force, modification, moment, root torque

## Abstract

**Objectives:**

Previous studies have demonstrated that aligners with labial‐cervical pressure points can induce root movement, but with initial unwanted tipping. This study assessed the impact of palatal‐incisal pressure points on improving root movement and reducing initial offset. Additionally, the influence of aligner materials on force and moment generation was evaluated.

**Material and Methods:**

The experimental setup consisted of an acrylic upper jaw model with teeth 11 and 21 separated and secured to a Hexapod using a 3D force‐moment sensor, allowing for the simulation of various malpositions of the measurement teeth. In addition to labial pressure points set close to the cervical margins at a depth of 1.5 mm, we investigated palatal pressure points positioned close to the incisal edge at depths ranging from 0.1 to 0.9 mm. We evaluated the force/moment (F/M) systems generated by both mono‐ and multi‐layered aligner materials during the simulated correction of 2° retroinclination of the measurement teeth. Five aligners were tested for each configuration. The relevant palatal torque range (palTR) was identified when the aligners simultaneously induced a negative palatal force (−Fy) and a negative palatal torque moment (−Mx).

**Results:**

PET‐G aligners without pressure points showed no effective torque range. In contrast, aligners with pressure points generated an effective torque range of an average of 1.02° ± 0.03° following initial tooth tipping. The palatal‐incisal pressure points showed a significant reduction or elimination of the initial offset. Our findings revealed a general correlation between palTR‐start displacement (initial offset range) and palatal pressure point depth (linear mixed‐effects models, *p* < 0.05). In this manner, the initial offset for the 0.6 mm pressure points was reduced by 81.1% compared to that of the unmodified aligners (from 1.57° to 0.3°).

**Conclusion:**

The addition of palatal‐incisal pressure points alongside labial‐cervical pressure points demonstrated a promising reduction in the initial offset range in an in vitro setting, potentially enhancing the efficiency of torque movement with aligners. However, further biomechanical and clinical studies are necessary for the clinical translation of these results.

## 1 Introduction

Over the past two decades, aligners have become popular tools for correcting tooth misalignments [1, 2, 3]. Initially, aligners were used as shape‐driven appliances mainly for mild malpositions in the front teeth [4, 5, 6]. However, advancements in aligner technology have enabled more complex tooth movements and expanded the therapeutic applications [6, 7, 8].

Among the most challenging tasks for aligners are bodily tooth movements and root torque, which rely on precise control of root movement [9, 10, 11, 12, 13]. To address these challenges and improve treatment outcomes, specific modifications to aligners have been suggested [9, 14, 15]. These modifications, as outlined by Sheridan et al., include attaching composite bumps to the crowns or reinforcing aligners with indentations in the dental model to enhance localised force application [16, 17, 18, 19]. These enhancements, including power ridges in the Invisalign system, apply targeted forces to facilitate controlled root movement [14, 15, 20]. From a biomechanical standpoint, these modifications create localised contact forces on the teeth, enhancing the force vector for movement and generating the necessary force couple for controlled root adjustments [9, 21]. However, the dynamic nature of tooth‐aligner interactions during treatment poses complexities as contact points shift throughout the process [11, 15].

There is ongoing debate over whether aligners can effectively produce controlled root movements for bodily tooth translation or true root torque [5, 22]. Existing studies have been inconsistent in clearly distinguishing between bodily tooth movement and crown tipping, or between changes in incisor inclination resulting from crown tipping and primary root torque. In the field of orthodontics, the concept of ‘torque’ originates from treatments involving multibracket appliances, where controlled root movement to alter a tooth's inclination typically involves twisting the arch wire around its longitudinal axis [23, 24, 25]. This action creates a force couple (or moment) within the bracket slot, which, combined with an appropriate force component, can potentially generate root torque [23, 24, 25]. It is important to emphasise that regardless of the appliance used, true root torque involves primarily moving the root while keeping the centre of the tooth crown relatively stable in its original vestibulo‐oral position. In contrast, predominant crown movements are associated with either controlled or uncontrolled tooth tipping.

A recent systematic review of bodily tooth movement using aligners revealed that aligners without modifications have limited potential to induce such movements [26]. Building on the findings of this review, a biomechanical study was conducted by our research group to explore various geometries of aligner pressure points and assess how these modifications affect the ability to torque central incisors with aligners [21]. The findings of our in vitro study indicated that aligners with particular modifications might produce sufficient palatally directed forces and torquing moments to support bodily movement or root torque of central incisors, at least within the parameters of our laboratory experiment. Another significant finding from this study was the initial movement of the tooth before reaching the necessary torquing moment. This kind of adverse movement necessitates further uprighting of the initially retroclined incisors to counterbalance the initial palatal tipping [21]. To improve the efficacy of aligners in achieving targeted tooth movements, we proposed the inclusion of an additional palatal pressure point in the aligner to address initial movement and prevent any initial misalignment during translation or torque actions.

The present biomechanical study assessed how a palatal pressure point influences the palatal root movement and reduction of offset. Furthermore, this study investigated the optimal depth for the palatal pressure point to achieve precise root torque movement while minimizing undesired palatal tipping. Additionally, this in vitro investigation evaluated the impact of different mono‐ and multi‐layer materials on the forces and moments involved in the palatal torque of central incisors.

## 2 Materials and Methods

### 2.1 Experimental Setup

**FIGURE 1:**
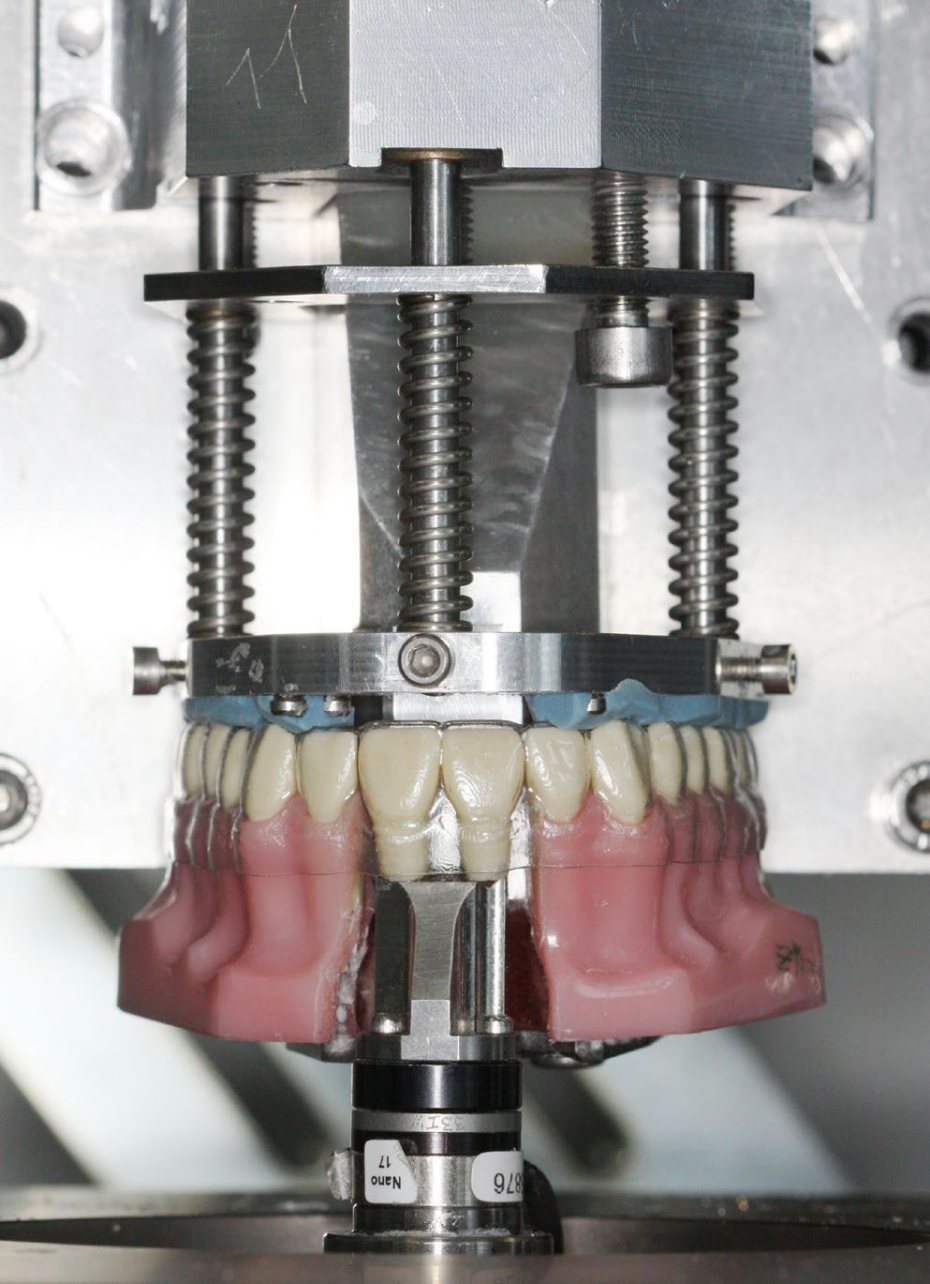
Detailed view of the test setup revealing measurement teeth 11 and 21 fixed to a 3D force‐moment sensor. A horseshoe‐shaped opposite jaw component with a silicone underlay (blue) secured the aligners during testing.

The experimental configuration involved an acrylic maxillary dentition model (Frasaco GmbH, Tettnang, Germany) with separated upper central incisors (teeth 11, 21) (Figure 1). These teeth were mounted on a three‐dimensional (3D) force and moment (F/M) sensor (Nano 17 Sensor; ATI Industrial Automation, Apex, USA) (Figure 1). A hexapod robot (PI M‐850; Physik Instrumente GmbH & Co. KG, Karlsruhe, Germany) was used to simulate various malpositions of teeth 11 and 21, while maintaining system rigidity. To prevent displacement of the aligners, a horseshoe‐shaped aluminium plate lined with dental silicone impression material (Silaplast Futur; Detax, Ettlingen, Germany) was used as an “aligner retaining device”. The entire experimental setup was housed in a temperature‐controlled chamber to maintain an ambient temperature of 37°C ± 0.5°C.

### 2.2 Aligner Modification Design

**FIGURE 2:**
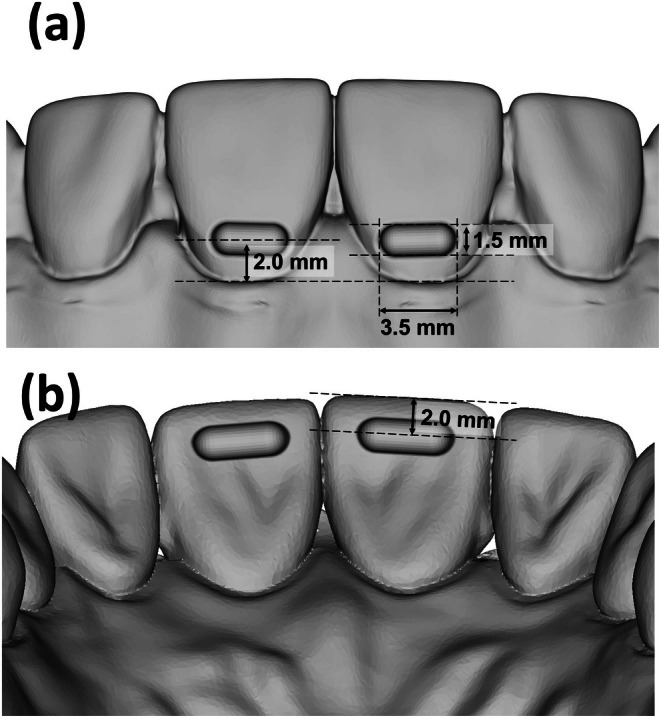
Screenshots of the digital model with the capsular pressure points inserted into both the labial‐cervical (a) and the palatal‐incisal tooth surfaces (b) of the measuring teeth 11, 21. These geometries were implemented at a distance of 2 mm from the gingival margin or 2 mm below the incisal edge. While the labial depth was uniformly kept at 1.5 mm for all test models, the palatal depth varied from 0.1 to 0.9 mm.

A 3D scan of the original maxillary Frasaco model was conducted using a desktop scanner (3Shape R2000, Copenhagen, Denmark) prior to the computer‐aided design of the various modifications. The examined geometry of the labial pressure points was 1.5 mm in height and 3.5 mm in width (Figure 2a), as described in our previous study [21]. Both pressure points were digitally designed and incorporated as indentations into the labial surface of teeth 11 and 21 using the OnyxCeph software (Image Instruments GmbH, Chemnitz, Germany), positioned 2 mm below the cervical margin.

In addition to the labial‐cervical pressure points on teeth 11 and 21 (Figure 2a), further pressure points at different depths were incorporated into the palatal surface of the measured teeth. These were placed at a distance of 2 mm from the incisal edge of teeth 11 and 22. The examined palatal‐incisal pressure point depths were 0.1, 0.2, 0.3, 0.6, and 0.9 mm (Figure 2b), and were also designed and positioned using OnyxCeph software. An additional model without palatal modifications was used for thermoforming the control aligners, resulting in six different digital test model configurations.

### 2.3 Aligner Selection and Fabrication

After digitally positioning the different pressure points, physical 3D models were printed for each digital model configuration using a DLP printer (Asiga Max, Asiga, Alexandria, Australia) and light‐curing resin (IMPRIMO LC Model, Scheu Dental GmbH, Iserlohn, Germany). All modifications were tested with three different aligner materials: conventional polyethylene terephthalate glycol (PET‐G) foil (Duran, Scheu Dental GmbH, Iserlohn, Germany) as a single‐layer material and CA Pro (Scheu Dental GmbH, Iserlohn, Germany) and Zendura FLX (Bay Materials LLC, Fremont, USA) as multilayer materials. The tested aligner thicknesses were 0.75 mm (Duran, CA Pro) and 0.76 mm (Zendura FLX). Five aligners were thermoformed for each modification configuration and aligner material, resulting in a total of 90 test aligners (6 configurations × 3 aligner materials × 5 samples).

### 2.4 Measurement Procedure

All tested aligners were acclimatised in the climate chamber for at least 30 min prior to testing to ensure a consistent temperature of 37°C. They were then moistened with artificial saliva (Glandosane, Cell Pharm, Bad Vilbel, Germany) directly before application to the acrylic maxillary dentition model. In this study, we simulated a 2° retroclination of teeth 11 and 21, replicating an Angle Class II/2 malocclusion. The aligner was initially positioned on the test model with the targeted teeth in the simulated 2° retroclined position. The simulated palatal root torque was then applied in precise 0.05° increments until the neutral position of teeth 11 and 21 was achieved. Mirroring orthodontic treatment with braces, the point of rotation was set at the midpoint of the imaginary line connecting the FA points of the measured teeth [11 and 21]. Correspondingly, the 3D F/M components were measured after each 0.05° torque movement increment. Each measurement was repeated three times with the same aligner. After each repetition, the aligner was removed and reseated on the test model.

### 2.5 Data Analysis

**FIGURE 3:**
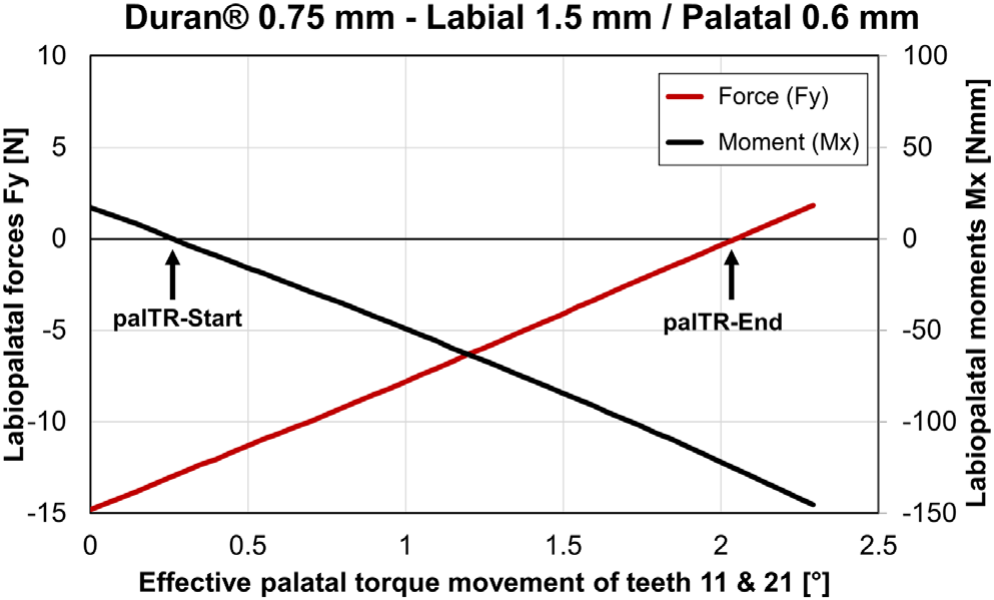
Labio‐palatal forces (Fy, red curve) and moments (Mx, black curve) measured during the simulated palatal torque movement of teeth 11 and 21 with the 0.75 mm Duran aligners, including 1.5 mm deep labial and 0.6 mm deep palatal capsular modifications. The black arrow marks the start of the effective palatal torque range (palTR‐start).

Data analysis was performed using individually developed algorithms programmed in MATLAB (MathWorks Inc., Natick, USA). The measured 3D F/M values were transferred to the estimated center of resistance of the measurement teeth (Cres), located at a distance of 13.8 mm from the incisal edge along the longitudinal axis of the root [27, 28]. This transformation of the 3D F/M resulted in individual F/M values directly corresponding to the induced 3D movements of tooth 11. For example, if all three moment components are zero, the tooth would undergo only a translation in the direction of the resultant force. Further data analysis was performed to assess the extent of induced palatal root torque movement. A schematic example of the corresponding F/M curves is presented in Figure 3. The biomechanical prerequisites for a palatal root torque of teeth 11 and 21 are both a palatally directed force (i.e., negative Fy) and a palatal root torqueing moment (i.e., negative Mx). The negative moment values (−Mx) cause the inclination of the teeth to change, resulting in labial tipping of the tooth crown. Therefore, translational movement with −Fy is also needed for the tooth crown to remain in place during palatal root torque movement. In this context, the zero crossing of the moments in the sagittal plane (Mx) with the x‐axis was defined as the start of the palatal torque range (palTR‐start). The end point was reached when Fy reached 0 N, indicating the end of the torque range (palTR‐end). The palatal torque range (palTR length) was defined as the distance between the start and end points. The Fy magnitudes at the palTR‐start and the labiopalatal moments Mx at the end (palTR‐end) were also extracted. Conversely, the lack of such a negative force (−Fy) and moment (−Mx) combination in a specific experimental data set indicated that the tested aligner was not capable of inducing bodily movement or root torque.

### 2.6 Statistical Analysis

Statistical analysis was performed using the statistical software R (R Foundation for Statistical Computing, Vienna, Austria). This included calculating the mean values and interquartile ranges for all relevant variables for the three measurement repetitions with the same aligner and the five aligners of each modification. To assess the impact of the two variables, palatal modification depth and aligner material, on the measured F/M values, palTR‐start, and palTR‐length, linear mixed‐effect models were employed. These models were calculated in two ways: separate models for each variable (modification depth or material) to analyse the effect of each variable individually and global models for each variable to examine the overall effect of each variable across all modification depths and materials. Furthermore, pairwise comparisons between the variables were conducted using the Wilcoxon‐Mann–Whitney test with a significance level of *α* = 0.05. To account for multiple comparisons, all *p*‐values were adjusted using the Holm‐Bonferroni method.

## 3 Results

Figure 3 presents the individual labiopalatal force (Fy) and moment (Mx) curves for the simulated palatal torque movement of teeth 11 and 21. These curves were obtained using 0.75 mm thick Duran aligners with specific modifications: 1.5 mm deep labial and 0.6 mm deep palatal capsular pressure points. The measured Fy/Mx curves exhibited an almost linear relationship with the simulated torque movement and were representative of all modified aligners across different foil types and palatal modification depths.

**FIGURE 4:**
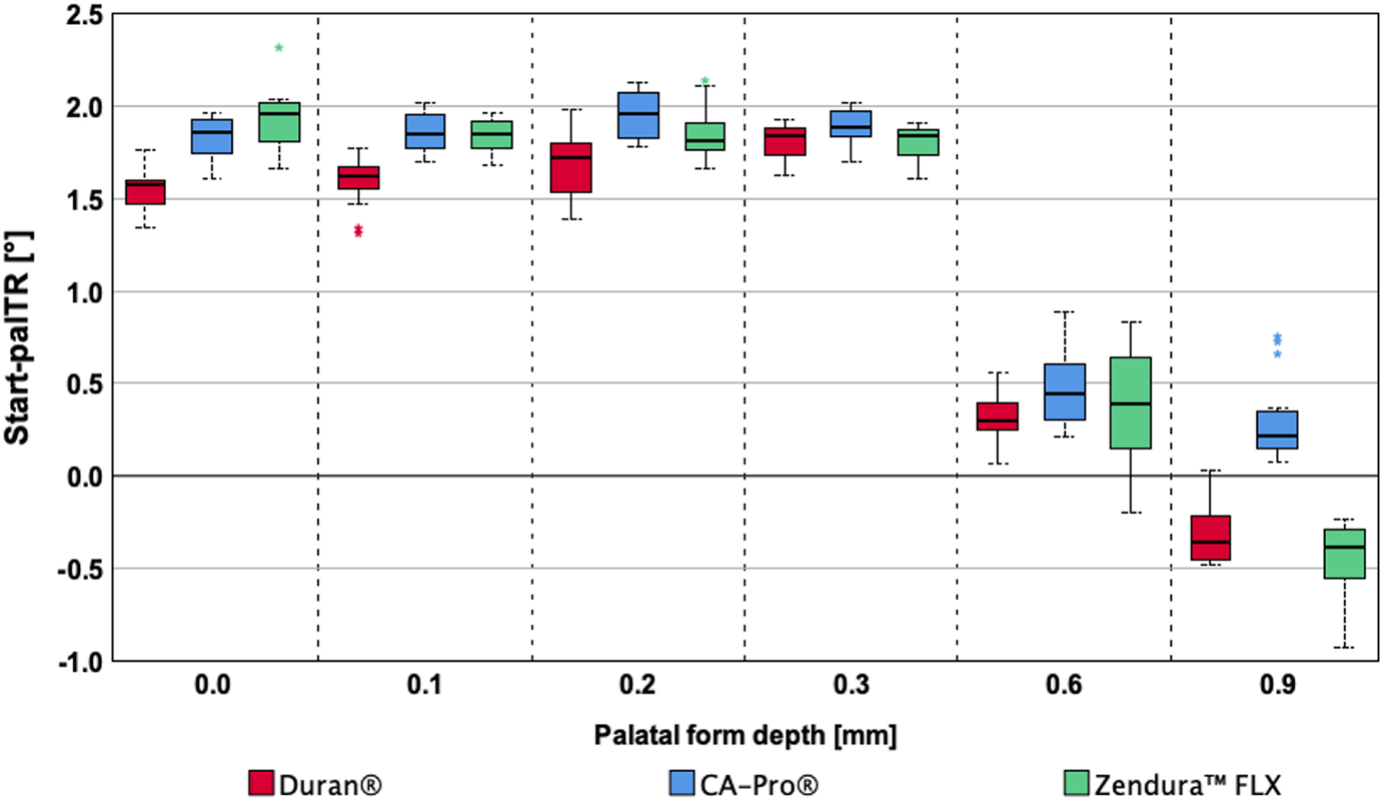
Effective palatal displacements [°] at which the palatal torque range started (palTR‐start). PalTR‐start values for 0.75 or 0.76 mm thick aligners with 1.5 mm deep labial and palatal capsular modifications in various depths (range 0–0.9 mm) integrated in three different foil types (CA Pro, Zendura FLX, and Duran) are shown.

At baseline (at the simulated malocclusion position), negative palatal force (Fy) magnitudes were measured, with a median value of approximately −14.54 N for this aligner configuration (Duran 0.75 and 0.6 mm deep palatal pressure points). However, the measured labiopalatal moments (Mx) in most aligners remained positive (directed labially). This resulted in palatal tipping of the measured teeth, indicating an initial inclination change in the opposite direction of the desired palatal root torque movement. For this modified variant (Figure 4), the median magnitude of Mx was 14.39 ± 8.04 Nmm. Notably, for some variants with 0.9 mm deep palatal pressure points, negative palatal Mx magnitudes were already measurable in the retroclined malposition of teeth 11 and 21.

During the experimental palatal root torque movement of teeth 11 and 21, all aligner modifications decreased the F/M components along the x‐axis. Interestingly, the labiopalatal forces (Fy) remained negative (approximately −7.5 N) even at the zero‐crossing point of the Mx curve. This zero‐crossing point, where the Mx curve intersects the x‐axis and changes polarity from positive to negative, marks the palTR‐start. Beyond this point, the conditions for successful palatal torque are met, with negative forces (−Fy) and negative moments (−Mx) acting on teeth 11 and 21. Notably, all variants achieved the required combination of negative Fy and Mx for the desired movement. Even at palTR‐end, the centre of resistance exhibited negative labiopalatal moments (approximately −70.4 Nmm). The palTR‐start displacements for all aligner foil types and modification depths are presented in Table 1. These results reveal a general correlation between palTR‐start displacement (initial offset range) and palatal pressure point depth (linear mixed‐effects models, *p* < 0.05).

**TABLE 1:** Initial palatal torque range (palTR‐start) and its width for various aligner foils, both with and without palatal capsular modifications.

Foil type and thickness	Modification shape	Form depth [mm]		Malposition (2° retroclination)		Palatal torque range (Mx = 0 Nmm)				
		Labial modification	Palatal modification	Fy (IQR) [*N*]	Mx (IQR) [*Nmm*]	Displacement palTR‐start (IQR) [°]	Fy palTR‐start (IQR) [*N*]	Width (IQR) [°]	Mx palTR‐end (IQR) [*Nmm*]	Stiffness (IQR) [Nmm/°]
Duran (monolayer) 0.75 mm	None	0.0	0.0	−9.50 (0.98)	57.89 (5.63)	1.32 (0.10)	−2.25 (0.69)	0.30 (0.10)	−24.40 (7.18)	82.18 (7.86)
	Capsular	1.5	0.0	−18.32 (4.89)	76.62 (11.40)	1.57 (0.13)	−9.53 (3.02)	1.13 (0.36)	−96.91 (28.09)	82.26 (4.33)
		1.5	0.1	−19.64 (3.17)	84.66 (14.02)	1.62 (0.12)	−9.86 (1.41)	1.17 (0.10)	−107.59 (14.78)	90.33 (4.99)
		1.5	0.2	−18.78 (6.18)	82.19 (10.79)	1.72 (0.26)	−8.74 (4.39)	1.26 (0.37)	−89.90 (43.30)	72.79 (10.59)
		1.5	0.3	−21.30 (4.93)	89.30 (14.76)	1.84 (0.14)	−10.41 (3.49)	1.26 (0.51)	−107.30 (30.24)	81.97 (5.70)
		1.5	0.6	−14.54 (1.87)	14.39 (8.04)	0.30 (0.15)	−12.67 (2.43)	1.72 (0.29)	−121.72 (16.99)	68.15 (3.27)
		1.5	0.9	−9.79 (2.15)	−30.70 (14.47)	−0.36 (0.24)	−13.57 (2.50)	1.68 (0.42)	−128.53 (27.63)	75.47 (8.97)
CA Pro (multilayer) 0.75 mm	None	0.0	0.0	−8.66 (0.94)	48.17 (5.78)	1.45 (0.25)	−2.04 (0.71)	0.27 (0.05)	−21.76 (8.23)	72.72 (30.15)
	Capsular	1.5	0.0	−8.97 (2.53)	43.29 (11.02)	1.86 (0.18)	−3.93 (1.43)	0.82 (0.21)	−41.97 (12.93)	50.70 (8.63)
		1.5	0.1	−10.12 (3.67)	51.48 (16.55)	1.85 (0.19)	−4.07 (2.17)	0.70 (0.33)	−41.13 (20.34)	54.29 (4.95)
		1.5	0.2	−10.49 (4.97)	52.00 (22.83)	1.96 (0.25)	−4.19 (2.40)	0.83 (0.30)	−41.45 (22.58)	50.16 (6.34)
		1.5	0.3	−12.36 (2.20)	60.60 (9.59)	1.89 (0.13)	−5.35 (1.04)	0.95 (0.15)	−53.13 (10.23)	55.39 (4.72)
		1.5	0.6	−8.76 (2.18)	19.04 (10.19)	0.45 (0.31)	−7.08 (2.07)	1.53 (0.25)	−64.55 (17.47)	39.26 (4.80)
		1.5	0.9	−4.65 (2.01)	7.31 (9.04)	0.21 (0.20)	−3.77 (2.08)	0.84 (0.41)	−37.51 (19.68)	43.46 (4.11)
Zendura FLX (multilayer) 0.76 mm	None	0.0	0.0	−7.39 (1.30)	41.44 (6.84)	1.70 (0.35)	−1.76 (0.89)	0.34 (0.04)	−17.50 (9.48)	52.47 (15.02)
	Capsular	1.5	0.0	−8.50 (2.89)	41.67 (21.61)	1.96 (0.20)	−3.24 (1.41)	0.77 (0.20)	−32.86 (13.58)	49.36 (16.84)
		1.5	0.1	−13.90 (0.60)	66.33 (2.95)	1.85 (0.14)	−6.18 (0.40)	0.99 (0.07)	−62.13 (4.98)	62.05 (3.16)
		1.5	0.2	−20.94 (12.72)	83.59 (42.46)	1.82 (0.15)	−10.80 (8.12)	1.68 (1.21)	−104.91 (76.86)	60.95 (8.22)
		1.5	0.3	−21.49 (1.37)	85.49 (3.02)	1.84 (0.14)	−11.32 (0.92)	1.79 (0.16)	−109.62 (8.66)	63.86 (2.68)
		1.5	0.6	−8.56 (5.26)	18.16 (22.24)	0.39 (0.49)	−5.54 (7.95)	1.25 (1.34)	−52.37 (67.36)	48.00 (6.93)
		1.5	0.9	−9.44 (0.61)	−21.15 (11.23)	−0.38 (0.26)	−11.67 (1.02)	2.17 (0.18)	−108.02 (5.46)	50.01 (1.79)

*Note:* The table includes corresponding force values (Fy) for the malposition (2° retroclination of teeth 11 and 21) and at palTR‐start, as well as the moments (Mx) at the conclusion of the palatal torque range (palTR‐end) and the stiffness of each aligner.

Abbreviation: IQR: interquartile range.

All palatal aligner modifications significantly reduced the initial offset area (Table 1). Differences of 0–0.3 mm in the depth were statistically insignificant and minimal (linear mixed‐effects models, *p* > 0.05). These variants exhibited starting points between 1.57° and 1.96° of simulated palatal displacement (Table 1). However, the positional change became statistically significant (*p* < 0.01) from the 0.6 mm depth onward. Here, the initial offset for the 0.6 mm pressure points (Table 1) was reduced by 81.1% compared to that of the unmodified aligners (1.57° to 0.3°). The 0.9 mm deep palatal modifications with Duran and Zendura FLX‐aligners eliminated the initial offset area (Table 1). These negative values (−0.36 mm) resulted from extrapolation, as the simulated malposition was already within the effective working range. Conversely, CA Pro‐aligners retained a minimal residual initial offset (0.21 mm).

Analysing the effective palatal torque range (palTR) (Table 1) revealed a distinct correlation between the width of the palTR and depth of the palatal pressure point (linear mixed‐effects models, *p* < 0.05). Accordingly, the aligners with the deepest modifications of 0.6 mm and 0.9 mm achieved the widest palTR, with median values of 1.5° and 1.56°, respectively.

The aligner material also influenced the palTR. The largest palTR (2.17 mm) was measured for the 0.9 mm modifications in Zendura FLX aligners (Table 1). However, these values were greater, and the palatal force (Fy) was lower for the 0.6 mm variant (−5.54 N) than for the Duran (−12.67 N) and CA Pro (−7.08 N) aligners. Overall, the Zendura FLX and Duran aligners demonstrated the widest ranges, with 1.72° for 0.6 mm Duran and 2.17° for 0.9 mm Zendura FLX. A median palatal root torque movement of 1.72° (Duran) corresponded to approximately half a millimetre of palatal displacement.

## 4 Discussion

Correcting the angulation of front teeth, particularly in Class II/2 malocclusions with retroclined incisors, presents a significant challenge for clear aligner therapy [26]. This movement, known as palatal torque, involves tipping the tooth root palatally while maintaining a relatively stable vestibulo–oral position of the crown. Achieving pure torque is difficult with aligners because they primarily exert force on the crown [9, 11, 21, 27].

To effectively induce palatal bodily movement or torque (palTR) using aligners, a strategic combination of two components, palatally directed force (−Fy) and an uprighting moment (−Mx), is essential to counteract the tipping tendency of palatally directed force [21]. Invisalign addresses this challenge by incorporating ‘power ridges’, which are pressure areas on the aligner's labio‐cervical region for counteracting unwanted crown‐tipping forces [14, 15, 26]. This approach was originally proposed by Sheridan et al. [16, 17, 18].

**FIGURE 5:**
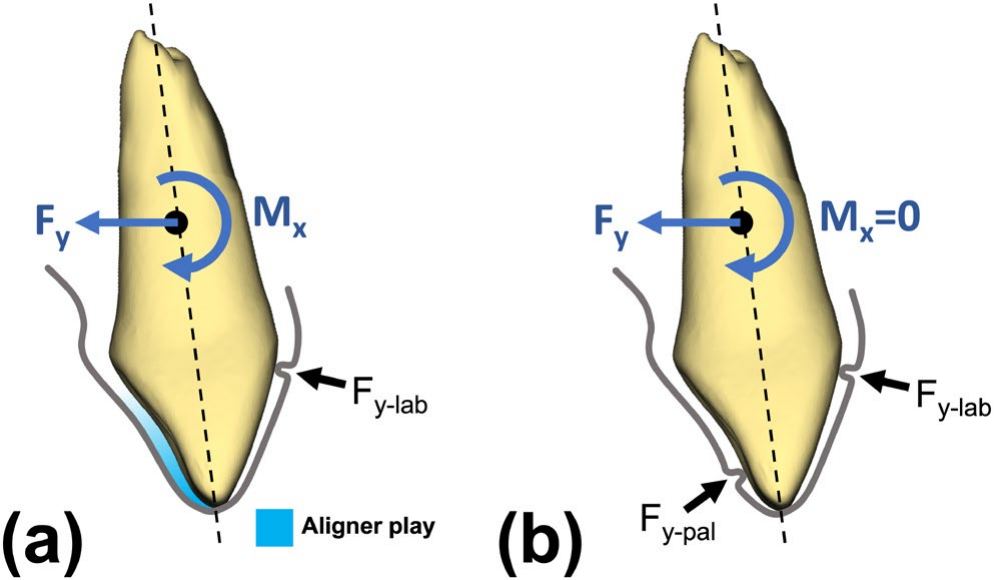
Schematic representation of the force‐moment (F/M) system generated by the aligners, highlighting the labio‐cervical pressure region and the initial aligner play. (a) The dominant labio‐cervical pressure point and the associated labial contact force (Fy‐lab) create palatal moments (Mx), resulting in initial tipping of the tooth. (b) Introduction of an additional contact force (Fpal) as a pressure point reduces or eliminates the initial aligner play, thereby mitigating tipping moments and potentially initiating the palatal torque range earlier.

Our previous in vitro study achieved palatal translation (movement toward the palate) of a single upper central incisor (tooth 11) using aligner modifications with labial pressure points [21]. This approach successfully generated the required forces (−Fy) and moments (−Mx) for the desired movement. However, a significant initial ‘offset range’ was observed before actual tooth movement began, causing the crown to tip palatally for a while before being uprighted (Figure 5a) [21]. This necessitates additional tooth movement to compensate for this tipping and achieve the desired uprighting of the root, potentially extending treatment duration [21].

Building on these findings, we conducted the current in vitro study investigating the palatal torque movement of two upper central incisors (teeth 11 and 21) using aligners with labial capsular modifications. To overcome the limitations of the offset range observed in the previous study and achieve more direct torque, an additional palatal pressure point was incorporated into the aligner design [21]. Moreover, we examined how varying the depth of these palatal pressure points and using different aligner materials (monolayer vs. multilayer) affects the forces and moments generated during stepwise simulated 2° palatal torque movement of both central incisors (Figure 5b).

Encouragingly, the results of this in vitro biomechanical study also demonstrated the feasibility, at least within an in vitro setting, of achieving pure palatal root movement (palatal torque) of two upper central incisors using modified aligners. The achievable range of palatal torque, between 0.7° and 2.3°, falls within a clinically relevant range for correcting mild to moderate cases of retroclined incisors.

In contrast to our previous study and the measurements with unmodified aligners, the addition of palatal pressure areas significantly reduced the initial offset range [21]. In some cases, these modifications even eliminated the offset entirely. In this manner, the initial palatal tipping prior to uprighting of the measurement teeth could be reduced, increasing the efficiency of the treatment. This improvement was likely because the palatal modifications generated the necessary force couple for palatal torque movement directly after seating the aligner.

The depth of the palatal pressure areas also emerged as a possible factor influencing the effectiveness of palatal torque. Our findings suggested that a depth exceeding 0.6 mm, ideally 0.6–0.9 mm, might be beneficial. This depth could generate a sufficient force in conjunction with existing labial forces. Compared to our previous study and unmodified aligners, Duran aligners with a 0.6 mm palatal pressure point demonstrated a reduction in the offset range. This finding provided preliminary evidence for the potential benefits of strategically placed palatal pressure points. Additionally, the results suggested that depths shallower than 0.3 mm might be inadequate. Based on the observed trends, incorporating palatal pressure points with depths exceeding 0.6 mm, ideally 0.6–0.9 mm, into the digital aligner design showed promise. While these findings offered a promising direction for improving palatal torque treatment, further controlled clinical trials are necessary to confirm the generalisability and long‐term effectiveness of this approach before widespread clinical use.

The study also focused on the influence of the palatal pressure point depth and aligner material on the width of the achievable palatal torque range. There was a positive correlation between the depth of the pressure point and the extent of the torque range. Aligners with the deepest modifications (0.6 and 0.9 mm) exhibited the widest ranges, with median values of 1.5° and 1.56°, respectively.

Compared with the Zendura FLX and CA Pro aligners, the Duran aligners were able to generate a sufficiently high negative force (−Fy). This difference was likely due to variations in aligner stiffness, with Duran being the least elastic material [29]. As a monolayer aligner material, Duran proved most reliable in our study for achieving efficient palatal root torque. It delivered a sufficiently long palatal torque range of approximately 1.7° with relatively low variability.

An important consideration when interpreting the results of this study is that the F/M values obtained were dependent on the types of aligners tested, including their cervical dimensions. Future research could explore whether aligners with a shorter cervical component can produce sufficient moment magnitudes for palatal root torque of the incisors [30, 31].

Another crucial aspect to consider is the viscoelastic behaviour of the different aligner materials. When combined with the limited velocity of tooth movement, this viscoelasticity can significantly reduce the F/M values exerted by the aligners, potentially compromising the overall efficiency of torque generation with aligners by up to 80% of the initially measured forces [32, 33, 34, 35, 36].

While this study focused on conventional thermoformed aligners, the findings may be applicable to 3D‐printed aligners as well. However, further research is necessary to directly compare the effectiveness of 3D‐printed aligners with strategically placed pressure points to that of conventional thermoformed materials, such as Duran [37]. In general, thermoformed aligners exhibit variability in pressure point depth due to material limitations during the thermoforming process and air pockets in the mould. In contrast, 3D printing may offer more consistent pressure point depths [37, 38].

It is important to acknowledge the limitations of an in vitro study when translating the results to clinical practice. A key limitation was the simulated nature of the palatal torque movement, which may not fully reflect real‐world scenarios. Although the starting point of the effective working range can be reliably estimated from these findings, the overall length of the range may be heavily influenced by the complexity of the actual tooth movement needed. A further limitation of our experimental setup, despite being optimised for maximum stiffness, was the possible residual compliance that may have affected the palTR‐start values [9]. Additionally, while we investigated the isolated movement of two teeth, clinical practice involves the simultaneous movement of multiple teeth. This can cause discrepancies between the aligner and adjacent teeth, leading to deformation of the aligner and potentially reduced forces applied by the investigated pressure areas. This interaction between adjacent teeth and the forces applied (F/M systems) creates a complex loading scenario similar to multibracket appliances. However, in vitro studies are valuable for exploring fundamental questions and controlling comparisons of materials and modifications.

In vitro studies, while valuable, inherently struggle to replicate the full complexity of the periodontal ligament. This tissue exhibits anisotropic mechanical behaviour, meaning that its resistance to forces varies depending on direction [39]. This characteristic undoubtedly plays a significant role in how teeth initially move, especially during palatal torque, where the aligner interacts with the periodontal ligament. To bridge this gap and gain a more realistic understanding of the effective working range length, a tooth movement model that incorporates this anisotropic behaviour is necessary. By simulating the F/M system within such a model, we could significantly improve the clinical relevance of these findings. Our research group is already exploring this avenue for further investigation.

## 5 Conclusion

Aligners incorporating integrated pressure points in the labio‐cervical and palatal regions demonstrate the potential to induce palatal root torque on maxillary central incisors. To optimise force delivery and eliminate initial aligner offset, a minimum palatal pressure point depth of 0.6 mm is recommended in conjunction with a 1.5 mm labio‐cervical pressure point. PET‐G aligner material exhibits superior force and moment transmission with consistent palatal torque generation compared to multi‐layered alternatives. Further clinical investigations are warranted to evaluate the efficacy of this approach in a clinical setting.

## Author Contributions

The authors confirm contribution to the paper as follows: Study conception and design: Fayez Elkholy, Bernd G. Lapatki, Falko Schmidt, and Rudolph Jäger. Data acquisition: Sophia Weber. Data Analysis: Sophia Weber, Fayez Elkholy, Rudolph Jäger, and Stephan Repky. Interpretation of data: Fayez Elkholy, Bernd G. Lapatki, and Falko Schmidt. Manuscript preparation: Fayez Elkholy. All authors reviewed the results and approved the final version of the manuscript.

## Ethics Statement

As this study biomechanically evaluated clear aligners using an in vitro test device and did not involve any human or animal subjects, ethical approval was not required.

## Conflicts of Interest

The authors declare no conflicts of interest.

## Data Availability

The authors have nothing to report.

## References

[1] Y. B. Acar , A. Kovan , M. Ateş , and S. Biren , “How Efficient Are Clear Aligners? Clear Aligners vs Traditional Orthodontic Treatment: A Systematic Review,” Turkish Journal of Orthodontics 27 (2014): 106–110, 10.13076/tjo-d-14-00016.

[2] M. O. Lagravere and C. Flores‐Mir , “The Treatment Effects of Invisalign Orthodontic Aligners: A Systematic Review,” Journal of the American Dental Association (1939) 136 (2005): 1724–1729, 10.14219/jada.archive.2005.0117. 16383056

[3] M. D. Rosvall , H. W. Fields , J. Ziuchkovski , S. F. Rosenstiel , and W. M. Johnston , “Attractiveness, Acceptability, and Value of Orthodontic Appliances,” American Journal of Orthodontics and Dentofacial Orthopedics 135 (2009): 276–282, 10.1016/j.ajodo.2008.09.020. 19268820

[4] H. D. Kesling , “The Philosophy of the Tooth Positioning Appliance,” American Journal of Orthodontics and Oral Surgery 31 (1945): 297–304, 10.1016/0096-6347(45)90101-3.

[5] G. Rossini , S. Parrini , T. Castroflorio , A. Deregibus , and C. L. Debernardi , “Efficacy of Clear Aligners in Controlling Orthodontic Tooth Movement: A Systematic Review,” Angle Orthodontist 85 (2015): 881–889, 10.2319/061614-436.1. 25412265 PMC8610387

[6] A. Papadimitriou , S. Mousoulea , N. Gkantidis , and D. Kloukos , “Clinical Effectiveness of Invisalign(R) Orthodontic Treatment: A Systematic Review,” Progress in Orthodontics 19 (2018): 37, 10.1186/s40510-018-0235-z. 30264270 PMC6160377

[7] N. D. Kravitz , B. Kusnoto , E. BeGole , A. Obrez , and B. Agran , “How Well Does Invisalign Work? A Prospective Clinical Study Evaluating the Efficacy of Tooth Movement With Invisalign,” American Journal of Orthodontics and Dentofacial Orthopedics 135 (2009): 27–35, 10.1016/j.ajodo.2007.05.018. 19121497

[8] V. D'Anto , R. Bucci , V. De Simone , L. Huanca Ghislanzoni , A. Michelotti , and R. Rongo , “Evaluation of Tooth Movement Accuracy With Aligners: A Prospective Study,” Materials (Basel) 15, no. 7 (2022): 2646, 10.3390/ma15072646. 35407978 PMC9000684

[9] F. Elkholy , T. Panchaphongsaphak , F. Kilic , F. Schmidt , and B. G. Lapatki , “Forces and Moments Delivered by PET‐G Aligners to an Upper Central Incisor for Labial and Palatal Translation,” Journal of Orofacial Orthopedics 76 (2015): 460–475, 10.1007/s00056-015-0307-3. 26446503

[10] D. K. Baldwin , G. King , D. S. Ramsay , G. Huang , and A. M. Bollen , “Activation Time and Material Stiffness of Sequential Removable Orthodontic Appliances. Part 3: Premolar Extraction Patients,” American Journal of Orthodontics and Dentofacial Orthopedics 133 (2008): 837–845, 10.1016/j.ajodo.2006.06.025. 18538247

[11] W. Hahn , A. Zapf , H. Dathe , et al., “Torquing an Upper Central Incisor With Aligners—Acting Forces and Biomechanical Principles,” European Journal of Orthodontics 32, no. 6 (2010): 607–613, 10.1093/ejo/cjq007. 20462912

[12] F. F. Dai , T. M. Xu , and G. Shu , “Comparison of Achieved and Predicted Tooth Movement of Maxillary First Molars and Central Incisors: First Premolar Extraction Treatment With Invisalign,” Angle Orthodontist 89 (2019): 679–687, 10.2319/090418-646.1. 30920875 PMC8111827

[13] X. J. Zhang , L. He , H. M. Guo , J. Tian , Y. X. Bai , and S. Li , “Integrated Three‐Dimensional Digital Assessment of Accuracy of Anterior Tooth Movement Using Clear Aligners,” Korean Journal of Orthodontics 45 (2015): 275–281, 10.4041/kjod.2015.45.6.275. 26629473 PMC4664903

[14] M. Simon , L. Keilig , J. Schwarze , B. A. Jung , and C. Bourauel , “Forces and Moments Generated by Removable Thermoplastic Aligners: Incisor Torque, Premolar Derotation, and Molar Distalization,” American Journal of Orthodontics and Dentofacial Orthopedics 145 (2014): 728–736, 10.1016/j.ajodo.2014.03.015. 24880843

[15] M. Simon , L. Keilig , J. Schwarze , B. A. Jung , and C. Bourauel , “Treatment Outcome and Efficacy of an Aligner Technique—Regarding Incisor Torque, Premolar Derotation and Molar Distalization,” BMC Oral Health 14 (2014): 68, 10.1186/1472-6831-14-68. 24923279 PMC4068978

[16] K. Hilliard and J. Sheridan , “Adjusting Essix Appliance at Chairside,” Journal of Clinical Orthodontics 34 (2000): 236.

[17] J. J. Sheridan , P. Armbruster , P. Nguyen , and S. Pulitzer , “Tooth Movement With Essix Mounding,” Journal of Clinical Orthodontics 38 (2004): 435–441. 15333960

[18] J. Sheridan , W. Ledoux , and R. Minn , “Essix Appliances: Minor Tooth Movement With Divots and Windows,” Journal of Clinical Orthodontics 20 (1994): 659–663.

[19] S. J. Bowman , “Improving the Predictability of Clear Aligners,” Seminars in Orthodontics 23 (2017): 65–75, 10.1053/j.sodo.2016.10.005.

[20] T. Castroflorio , F. Garino , A. Lazzaro , and C. Debernardi , “Upper‐Incisor Root Control With Invisalign Appliances,” Journal of Clinical Orthodontics 47 (2013): 346–351. 23863556

[21] F. Elkholy , S. Weber , S. Repky , R. Jager , F. Schmidt , and B. G. Lapatki , “Are Aligners Capable of Inducing Palatal Bodily Translation or Palatal Root Torque of Upper Central Incisors? A Biomechanical In Vitro Study,” Clinical Oral Investigations 27 (2023): 4289–4300, 10.1007/s00784-023-05046-7. 37243819 PMC10415518

[22] G. Djeu , C. Shelton , and A. Maganzini , “Outcome Assessment of Invisalign and Traditional Orthodontic Treatment Compared With the American Board of Orthodontics Objective Grading System,” American Journal of Orthodontics and Dentofacial Orthopedics 128 (2005): 292–298, 10.1016/j.ajodo.2005.06.002. 16168325

[23] V. Jayade , S. Annigeri , C. Jayade , and P. Thawani , “Biomechanics of Torque From Twisted Rectangular Archwires. A Finite Element Investigation,” Angle Orthodontist 77 (2007): 214–220, 10.2319/0003-3219(2007)077[0214:BOTFTR]2.0.CO;2. 17319754

[24] E. D. Rauch , “Torque and Its Application to Orthodontics,” American Journal of Orthodontics 45 (1959): 817–830, 10.1016/0002-9416(59)90222-2.

[25] A. Archambault , R. Lacoursiere , H. Badawi , P. W. Major , J. Carey , and C. Flores‐Mir , “Torque Expression in Stainless Steel Orthodontic Brackets. A Systematic Review,” Angle Orthodontist 80 (2010): 201–210, 10.2319/080508-352.1. 19852662 PMC8978750

[26] S. L. Weber , G. Bernd , and F. Elkholy , “Labiopalatal Bodily Movement and Torque Control of Incisors With Aligners: A Systematic Review,” Journal of Aligner Orthodontics 7 (2023): 13–23.

[27] F. Elkholy , F. Schmidt , R. Jager , and B. G. Lapatki , “Forces and Moments Delivered by Novel, Thinner PET‐G Aligners During Labiopalatal Bodily Movement of a Maxillary Central Incisor: An In Vitro Study,” Angle Orthodontist 86 (2016): 883–890, 10.2319/011316-37R.1. 27224904 PMC8597333

[28] M. E. Geiger and B. G. Lapatki , “Locating the Center of Resistance in Individual Teeth via Two‐ and Three‐Dimensional Radiographic Data,” Journal of Orofacial Orthopedics 75 (2014): 96–106, 10.1007/s00056-013-0198-0. 24577014

[29] B. Golkhani , A. Weber , L. Keilig , S. Reimann , and C. Bourauel , “Variation of the Modulus of Elasticity of Aligner Foil Sheet Materials due to Thermoforming,” Journal of Orofacial Orthopedics 83 (2022): 233–243, 10.1007/s00056-021-00327-w. 34414481 PMC9225978

[30] X. Lyu , X. Cao , J. Yan , R. Zeng , and J. Tan , “Biomechanical Effects of Clear Aligners With Different Thicknesses and Gingival‐Margin Morphology for Appliance Design Optimization,” American Journal of Orthodontics and Dentofacial Orthopedics 164 (2023): 239–252, 10.1016/j.ajodo.2022.12.014. 36935221

[31] T. M. Elshazly , L. Keilig , D. Salvatori , P. Chavanne , M. Aldesoki , and C. Bourauel , “Effect of Trimming Line Design and Edge Extension of Orthodontic Aligners on Force Transmission: An In Vitro Study,” Journal of Dentistry 125 (2022): 104276, 10.1016/j.jdent.2022.104276. 36055460

[32] F. Elkholy , S. Schmidt , F. Schmidt , M. Amirkhani , and B. G. Lapatki , “Force Decay of Polyethylene Terephthalate Glycol Aligner Materials During Simulation of Typical Clinical Loading/Unloading Scenarios,” Journal of Orofacial Orthopedics 84 (2023): 189–201, 10.1007/s00056-021-00364-5. 34882259 PMC10119250

[33] L. Lombardo , E. Martines , V. Mazzanti , A. Arreghini , F. Mollica , and G. Siciliani , “Stress Relaxation Properties of Four Orthodontic Aligner Materials: A 24‐Hour In Vitro Study,” Angle Orthodontist 87 (2017): 11–18, 10.2319/113015-813.1. 27314603 PMC8388588

[34] L. R. Iwasaki , J. E. Haack , J. C. Nickel , and J. Morton , “Human Tooth Movement in Response to Continuous Stress of Low Magnitude,” American Journal of Orthodontics and Dentofacial Orthopedics 117 (2000): 175–183, 10.1016/s0889-5406(00)70229-0. 10672218

[35] A. Dudic , C. Giannopoulou , and S. Kiliaridis , “Factors Related to the Rate of Orthodontically Induced Tooth Movement,” American Journal of Orthodontics and Dentofacial Orthopedics 143 (2013): 616–621, 10.1016/j.ajodo.2012.12.009. 23631963

[36] M. Amirkhani , F. Elkholy , and B. G. Lapatki , “Clear Aligners: Material Structures and Properties,” in Principles and Biomechanics of Aligner Treatment, ed. R. Nanda , T. Castroflorio , F. Garino , and K. Ojima (Elsevier, 2022), 30–34.

[37] G. M. Tartaglia , A. Mapelli , C. Maspero , et al., “Direct 3D Printing of Clear Orthodontic Aligners: Current State and Future Possibilities,” Materials (Basel) 14, no. 7 (2021): 1799, 10.3390/ma14071799. 33916462 PMC8038630

[38] J. Grant , P. Foley , B. Bankhead , G. Miranda , S. M. Adel , and K. B. Kim , “Forces and Moments Generated by 3D Direct Printed Clear Aligners of Varying Labial and Lingual Thicknesses During Lingual Movement of Maxillary Central Incisor: An In Vitro Study,” Progress in Orthodontics 24 (2023): 23, 10.1186/s40510-023-00475-2. 37423974 PMC10329968

[39] K. Papadopoulou , I. Hasan , L. Keilig , et al., “Biomechanical Time Dependency of the Periodontal Ligament: A Combined Experimental and Numerical Approach,” European Journal of Orthodontics 35 (2013): 811–818, 10.1093/ejo/cjs103. 23314330

